# Accumulated coercion and short-term outcome of inpatient psychiatric care

**DOI:** 10.1186/1471-244X-10-53

**Published:** 2010-06-28

**Authors:** Lars Kjellin, Tuula Wallsten

**Affiliations:** 1 School of Health and Medical Sciences, Psychiatric Research Centre, Örebro University, Örebro, Sweden; 2 Centre for Clinical Research, Uppsala University, Central Hospital, Västerås, Sweden

## Abstract

**Background:**

The knowledge of the impact of coercion on psychiatric treatment outcome is limited. Multiple measures of coercion have been recommended. The aim of the study was to examine the impact of accumulated coercive incidents on short-term outcome of inpatient psychiatric care

**Methods:**

233 involuntarily and voluntarily admitted patients were interviewed within five days of admission and at discharge or after maximum three weeks of care. Coercion was measured as number of coercive incidents, i.e. subjectively reported and in the medical files recorded coercive incidents, including legal status and perceived coercion at admission, and recorded and reported coercive measures during treatment. Outcome was measured both as subjective improvement of mental health and as improvement in professionally assessed functioning according to GAF. Logistic regression analyses were performed with patient characteristics and coercive incidents as independent and the two outcome measures as dependent variables

**Results:**

Number of coercive incidents did not predict subjective or assessed improvement. Patients having other diagnoses than psychoses or mood disorders were less likely to be subjectively improved, while a low GAF at admission predicted an improvement in GAF scores

**Conclusion:**

The results indicate that subjectively and professionally assessed mental health short-term outcome of acute psychiatric hospitalisation are not predicted by the amount of subjectively and recorded coercive incidents. Further studies are needed to examine the short- and long-term effects of coercive interventions in psychiatric care.

## Background

The worldwide use of coercion in mental health services is based on the assumption that coercion in specific situations will lead to a better outcome, in terms of prevention of danger to self or others and health improvement, than if the patients were not coerced into treatment. Still, the knowledge of the impact of coercion on outcome is limited [1]. Katsakou & Priebe [2] found in a review of 18 outcome studies that most involuntarily admitted patients show substantial clinical improvement, and that retrospectively between 33 and 81% found the admission as justified and/or the treatment as beneficial. They conclude that it is not possible to determine whether the differences in results reflect true differences or different methodologies, and that data on predictors of outcomes is limited.

In a subsequent Swedish study [3], predictors for a positive subjective short-term health outcome of short-term inpatient psychiatric care were found to be patients' self-reports of being well treated by the staff and having specific contact persons among the staff at the ward. Predictors for an improved functioning, assessed by professionals, were low baseline level of functioning and having a mood disorder diagnosis. Coercion at admission and the occurrence of coercive treatment or coercive measures during care, however, were not associated neither to subjective, nor to assessed, short-term outcome of inpatient psychiatric care. Coercion at admission was measured on the one hand as formal legal status (involuntary/voluntary), and on the other as perceived coercion according to the MacArthur Perceived Coercion Scale, MPCS [4] and to the Coercion Ladder, CL [5,6]. Coercion during care was measured according to patients' self-reports, only.

Legal status at admission is, however, a limited measure of coercion. The subsequent inpatient stay after an involuntary hospitalization may vary considerably in length, and in Sweden, like in many countries, formally voluntarily admitted patients may later on during the inpatient care episode be involuntarily detained. Perceived coercion at admission includes the initial experiences of the patient, only. The experiences of forced treatment, of being restrained, and of other coercive measures during treatment are likely to influence the total burden of being coerced. Iversen et al [7] found that accumulated coercive events predicted lessened patient satisfaction, and recommend multiple measures of coercion to be used in future studies.

Consequently, the aim of this article is to study the impact of accumulated coercive incidents on short-term outcome of inpatient psychiatric care.

## Methods

A consecutive sample of involuntarily admitted patients, and voluntarily admitted patients who were later on involuntarily detained, and a random sample of voluntarily admitted patients, at acute wards in four Swedish psychiatric services during specified periods of time in 1997-1999, were included in the study. Excluded were i) patients with main diagnoses of substance abuse, ii) forensic patients, iii) patients who did not speak Swedish or for other reasons were unable to carry through the interview, iv) patients not living in the catchment areas of the included services, and v) patients readmitted during the study periods and already included.

### Assessments

At a first interview within five days of admission, a structured patient interview was performed using the Nordic Admission Interview protocol [6]. It includes the CL, a visual analogue scale shown to the patients who were asked to mark their degree of perceived coercion at admission whilst instructions were read. Scores range from 1-10, where 10 represent the highest degree of perceived coercion. Scores ≥ 5 were regarded as high perceived coercion [3,8]. The patients were diagnosed according to DSM-IV [9], and the diagnoses were classified into psychoses (schizophrenia, delusional, schizoaffective, and schizophreniform disorders, and atypical psychoses), mood disorders (including bipolar disorders), and other diagnoses. Social functioning was assessed according to the Global Assessment of Functioning scale, GAF [9], and psychiatric symptoms according to the Brief Psychiatric Rating Scale, BPRS [10], with 16 items scoring from 0-6.

A structured follow-up interview was conducted at discharge or after three weeks of care, if the patient was not discharged by then. In the present study, a question concerning experiences of measures against the patient's own will at the ward was used to indicate perceived coercion during treatment.

The interviewers were psychiatrists and psychiatric psychologists, social workers or nurses, trained in interviewing and not personally involved in the treatment of the included patients. The interrater reliability has been found to be good, with a Cohen's kappa of 0.96 [11]. Additional data, including the occurrence of coercive treatment and coercive measures during inpatient care, were collected from the case records. The study was approved by the Medical Research Ethics Committee of the University of Uppsala.

### Measures of coercion and outcome

Coercion was measured as number of coercive incidents (CI):

- Involuntary legal status at admission or voluntarily admitted and subsequently involuntarily detained (= 1 CI)

- High perceived coercion at admission (CL ≥ 5 = 1 CI)

- Answers to the follow-up interview question "Have you been subjected to measures against your own will during this treatment period?" (yes, once = 1 CI, yes, occasionally = 2 CI, yes, repeatedly = 3 CI)

- The occurrence of forced medication, restraint by belt, and seclusion during treatment (each = 1 CI) according to the case record

Thus, CI could range from 0-8 subjective and recorded events for each patient.

Two outcome measures were used:

A. Subjective outcome. In the follow-up interview, the patients were asked: "Considering your mental problems, how do you feel now compared with at the time of admission?" Answers were classified as improved or not improved.

B. Assessed outcome. An increase in GAF scores ≥ 10 was considered as an improvement. To ensure robust findings, we transformed GAF change into a dichotomous outcome. A cut-off of 10 was regarded as a clinically significant change, and an interrater reliability test previously performed within the project [12] indicated that a change of 10 scores would not be due to differences in ratings.

### Statistics

The Chi-square test was used to test for differences in proportions. For differences in age, the t-test was used, and for differences in GAF, BPRS, and coercive incidents the Mann-Whitney U test. Logistic regression analyses were performed with sex, age, diagnosis, GAF, BPRS, and CI as independent and the two outcome measures as dependent variables. P-values < 0.05 were considered significant.

### Patients

Of 375 patients, asked to participate in the study, 282 consented. The patients who did not want to participate did not differ in gender or age from the participating patients. At follow-up, 235 patients were interviewed. Due to missing data regarding subjective outcome, a total of 233 patients were included in the study. Of these, 101 were involuntarily admitted, 16 voluntarily admitted and subsequently involuntarily detained, and 116 legally voluntarily admitted throughout the treatment period. Patient characteristics are presented in Table 1. "Other diagnoses" were mainly anxiety disorders and personality disorders.

**Table 1:** Characteristics of included patients

	Involuntarily admitted or detained patients	Voluntarily admitted patients	Total
	**n = 117**	**n = 116**	**n = 233**

**Sex**			

Men	43.6%	27.6%	35.6%

Women	56.4%	72.4%	64.4%

**Age**			

Mean(sd)	41.3(12.9)	42.0(12.0)	41.6(12.5)

**Diagnosis**			

Psychoses	50.0%	19.0%	34.5%

Mood disorders	31.9%	44.8%	38.4%

Other diagnoses	18.1%	36.2%	27.2%

**GAF score**			

Mean(sd)	34.3(10.7)	41.4(11.8)	37.9(11.8)

**BPRS score**			

Mean(sd)	29.1(13.7)	24.6(9.6)	26.7(12.0)

## Results

Of the 233 included patients, 67% were subjectively improved and 33% not improved. According to GAF, 58% were improved and 42% not improved (GAF data at follow-up was missing in eleven cases). Thirty-seven percent had no coercive incidents, 11% one coercive incident, 14% two incidents, 10% three incidents, 9% four incidents, 10% five incidents, 7% six incidents, and 2% seven incidents. None had eight coercive incidents (data on CI were missing in 15 cases). The mean(sd) number of coercive incidents was 2.1(2.1), and the median was 2.0. There were no differences in CI between those improved and those not improved, neither subjectively nor assessed according to GAF (Table 2).

**Table 2:** Accumulated coercion and subjective and assessed short-term outcome

Coercive incidents	Subjective outcome		Assessed outcome (GAF change)	
	
	Improved	Not Improved	Improved	Not Improved
	
	n = 151	n = 67	n = 121	n = 88
Mean	2.1	2.2	2.3	1.9

SD	2.1	2.3	2.3	2.0

Median	2.0	1.0	2.0	1.0

Min	0	0	0	0

Max	7	7	7	6

There were no differences in gender, age, diagnosis, GAF or BPRS scores between subjectively improved and not improved patients. Neither did improved patients differ in gender or age from those not improved when assessed by change in GAF, but the distribution on diagnostic groups differed. Of improved patients, 36% were classified as psychoses, 43% as mood disorders and 20% other diagnoses. Corresponding figures for not improved patients were 34, 29, and 37%, respectively (Chi2 = 8.53, df = 2, p = 0.014). Furthermore, assessed improved patients had lower GAF (mean[sd] 34[9] vs. 43[12], z = -5.65, p < 0.001) and higher BPRS scores (mean[sd] 29[12] vs.24 [12], z = -3.49, p < 0.001) at admission than not improved patients.

In the logistic regression analyses, gender, age, diagnosis, GAF and BPRS scores, and CI categorized in three groups (0, 1-3, and 4-7 CI) were entered as independent variables and the two outcome measures as dependent variables. Subjective improvement was not found to be predicted by coercive incidents (Table 3). Neither was assessed improvement according to GAF predicted by CI (Table 4). Patients classified as having "other diagnoses" were less likely to be subjectively improved, and a low GAF at admission predicted an improvement in GAF. Furthermore, there were tendencies of patients with mood disorders and patients with high BPRS scores at admission being more likely to improve in GAF scores.

**Table 3:** Logistic regression with subjective short-term outcome as dependent variable

Independent variables	OR	95% C.I.	p
Gender			

Female	1.00		

Male	1.08	0.56-2.08	0.817

			

Age	1.01	0.98-1.03	0.722

			

Diagnosis			

Psychoses	1.00		

Mood disorders	0.61	0.28-1.33	0.213

Other diagnoses	0.33	0.14-0.96	**0.010**

			

Baseline GAF	0.99	0.97-1.02	0.644

			

Baseline BPRS score	0.99	0.96-1.02	0.407

			

Coercive incidents			

0	1.00		

1-3	1.43	0.66-3.08	0.363

4-7	0.68	0.32-1.48	0.332

**Table 4:** Logistic regression with short-term change in GAF scores as dependent variable

Independent variables	OR	95% C.I.	p
Gender			

Female	1.00		

Male	1.26	0.64-2.46	0.507

			

Age	0.98	0.96-1.01	0.189

			

Diagnosis			

Psychoses	1.00		

Mood disorders	2.18	0.99-4.80	0.052

Other diagnoses	0.81	0.34-1.91	0.626

			

Baseline GAF	0.92	0.89-0.96	**< 0.001**

			

Baseline BPRS score	1.02	0.996-1.05	0.099

			

Coercive incidents			

0	1.00		

1-3	0.53	0.24-1.15	0.107

4-7	0.65	0.29-1.49	0.309

## Discussion

As in our previous study [3], based on the same material, coercion was not related to short-term outcome of psychiatric inpatient treatment, even though a measure more likely to capture the amount of coercion experienced by the patients was used in the present study. Diagnoses and level of functioning at admission were the only significant outcome predictors. A floor effect for the less healthy patients was indicated by the fact that patients with lower GAF and higher BPRS scores at admission were more likely to be improved in GAF scores.

A follow-up time of maximum three weeks is short but still relevant. The number of psychiatric beds in Sweden has been reduced and lengths of stay have been shortened [13,14]. Psychiatric hospital beds have to be used as effective as possible in order to help patients in acute phases of mental illness being able to rely on outpatient services in the community. Thus, it is important to measure mental health outcome after short periods of inpatient stay. We have used subjective as well as professionally assessed measures of coercion since many studies have shown great inconsistencies between perceived coercion and legal status at admission [7,8,15-18], and between self-reported and recorded coercive measures during inpatient treatment [19-21]. Since the time of our data collection, there has been a further decrease of psychiatric hospital admission rates in Sweden, while the numbers of involuntarily admitted patients have been quite stable. Consequently, the quota of involuntarily admitted patients in psychiatric inpatient care has increased. On a census day in 1997, 30% of all psychiatric inpatients were involuntarily admitted (forensic patients included), compared to 44% on a census day in 2008 [22]. Further studies are needed to examine whether these changes might have an impact on our findings.

We were inspired to do the analyses of the present study by Iversen et al [7]. It seems reasonable to assume that the total amount of coercion a person is subjected to is more likely to affect outcome than a single measure of coercion, only. However, while they found that accumulated coercion predicted lessened patient satisfaction, we found no association between accumulated coercion and subjective or assessed outcome. A probable explanation is that measures of coercion were different in the two studies, and that outcome, as measured in the present study, and patient satisfaction are quite different aspects. Furthermore, the legal definitions of coercive treatment may differ. According to the Swedish Compulsory Psychiatric Care Act all injections of involuntarily hospitalised patients should be recorded as coercive, but not orally given medication, while Iversen et al seem to include both oral medication and injections in the concept "involuntarily administered medication".

Previous studies, too, on coercion and outcome [23-27] have shown contradictory results, probably influenced by differing legal prerequisites in different countries, differing inclusion criteria, and different measures and methodologies. Despite the serious nature of involuntary hospitalisation and treatment of persons with mental illnesses, the effects of coercive interventions in mental health services are still not known. Large-scale studies with uniform methods allowing for analyses of sub-samples and controlling for differences in patient and treatment characteristics are called for.

Even less is known about the long-term outcome of involuntary admissions to psychiatric hospitals. In a unique study with one year follow-up, Priebe et al [28] found that patients' views of treatment within the first week are a relevant indicator for the long-term prognosis of involuntarily admitted patients. To study the impact of coercion, however, also voluntarily admitted patients need to be included in prospective studies, and as Høyer [1] has pointed out, judged by face validity the total number of days of deprivation of liberty seems to be a more adequate measure of coercion than formal legal status of the patient at admission.

Outcome was in our study measured as subjective and assessed improvement. As to the latter, a difference of 10 could be regarded as clinically more significant in lower than in higher GAF scores. However, 80% of the patients had GAF scores below 50. We were not able to assess outcome in terms of prevention of dangerous acts against self or others. Occasional randomised studies of outpatient commitment have been performed [29], but like in all other studies of involuntary hospitalisation, an inability to assign patients randomly to either compulsory or voluntary treatment is a limitation in the present study. RCT-studies of involuntary psychiatric treatment are, however, hardly possible to perform for ethical, legal and practical reasons. The studied sample in our study is not representative for all psychiatric inpatients, and the exclusion criteria applied may have biased the results. Furthermore, there were dropouts at inclusion and at follow-up, but considering that the patients were acutely mentally ill persons the dropout rate was acceptable.

## Conclusions

The present study indicates that subjectively and professionally assessed mental health short-term outcomes of acute psychiatric hospitalisation are not predicted by the amount of subjectively and "objectively" recorded coercive incidents patients are exposed to. Further studies are needed to examine the short- and long-term effects of coercive interventions by mental health services for different patient categories and with different preconditions.

## Competing interests

The authors declare that they have no competing interests.

## Authors' contributions

The study is part of the Swedish Coercion Study, which was supervised by LK and in which TW participated in the data collection. LK and TW both did the data analyses for this article. LK drafted and finalised the manuscript. TW read and commented and added to the drafts. Both authors read and approved the final manuscript.

## Pre-publication history

The pre-publication history for this paper can be accessed here:

http://www.biomedcentral.com/1471-244X/10/53/prepub
